# Mediating effect of family functioning on depressive symptoms and family resilience in dementia caregivers

**DOI:** 10.3389/fpubh.2026.1784746

**Published:** 2026-05-11

**Authors:** Xinyi Liu, Jiayi Li, Jun Yao, Li Zhang

**Affiliations:** 1 School of Health Economics and Management, Nanjing University of Chinese Medicine, Nanjing, Jiangsu, China; 2 School of Health Policy and Management, Nanjing Medical University, Nanjing, Jiangsu, China

**Keywords:** caregiver, dementia, family functioning, family resilience, mediation effect

## Abstract

**Background:**

Caregivers of individuals with dementia often assume substantial responsibilities and are vulnerable to mental health conditions such as depression, which may undermine both family resilience and overall family functioning.

**Objective:**

This study aimed to examine the association between depressive symptoms and family resilience among dementia caregivers, and to explore the potential mediating role of family functioning.

**Methods:**

A cross-sectional survey was conducted among 514 community-based caregivers of individuals with dementia. Participants completed the Self-Rating Depression Scale (SDS), the Family Resilience Assessment Scale (FRAS), and the General Functioning subscale of the Family Assessment Device (FAD-GF). Partial correlation analyses were used to assess the associations among key variables, and the bootstrap method was employed to evaluate the mediating effect of family functioning.

**Results:**

The mean family resilience score among caregivers was 99.78 ± 16.09. Family resilience was negatively correlated with depressive symptoms (*r* = −0.399, *p* < 0.001) and family functioning scores (*r* = −0.617, *p* < 0.001), while family functioning scores were positively correlated with depressive symptoms (*r* = 0.330, *p* < 0.001). Mediation analysis indicated that family functioning partially mediated the association between depression and family resilience, accounting for 44.39% of the total effect.

**Conclusion:**

Family functioning mediates the impact of depression on family resilience. Interventions aimed at improving family functioning may help strengthen family resilience in caregivers of individuals with dementia.

## 1 Introduction

Dementia is a progressive neurodegenerative syndrome characterized by a decline in cognitive function, memory, and the ability to perform activities of daily living (1). With the rapid aging of the population in China, the prevalence of dementia has risen markedly in recent years, reaching approximately 16.99 million in 2021 and is projected to rise to 22.2 million by 2030 (2). The disease is typically prolonged in course, and during the middle to late stages, individuals with dementia progressively lose the capacity for self-care and become entirely dependent on others for their daily needs.

In China, over 80% of individuals with dementia rely on informal, long-term care provided primarily by family members (3). The stress levels experienced by these family caregivers are reported to be approximately three times higher than those of the general population not engaged in caregiving (4).

Despite limited knowledge of dementia, restricted access to professional support services, and insufficient caregiving skills or confidence in their ability to manage care effectively, most caregivers often provide sustained, daily care over extended periods (5, 6). These challenges contribute to substantial psychological stress, with many caregivers experiencing persistent negative emotional states, including depression and anxiety. This not only increases the caregiving burden but also negatively impacts the quality of life of individuals with dementia under their care (7). Studies have shown that caregivers of dementia experience significantly higher levels of stress, anxiety, and depression than caregivers of other chronic diseases (8). This is not only due to the physical burden of caregiving tasks but also to the continued decline in patients’ cognitive function and unpredictable neuropsychiatric symptoms (9). Depression is the most common psychopathological outcome among caregivers. It significantly reduces the quality of life of caregivers, decreases care efficiency, increases the risk of inappropriate caregiving, and accelerates patient admission to nursing homes, thus placing enormous pressure on the broader social care system (10). Because informal care occurs within the family system, the emotional distress experienced by caregivers extends beyond the individual level and affect broader family processes.

Family resilience, defined as the capacity of a family system to mobilize resources and recover quickly in the face of crises and stress, serves as a key theoretical framework for understanding caregiver adaptation (11). In the context of long-term care stress, family resilience reflects the ability of families to restructure roles, maintain emotional connections and maintain adaptive functioning in the face of ongoing challenges (12). Within this framework, as cumulative stress factors increase, individuals within a family may develop or exacerbate depressive symptoms, placing an excessive burden on the family system (13). Empirical studies have shown that there is a dynamic relationship between family resilience and mental health (14). Depressive symptoms may disrupt communication patterns, weaken emotional cohesion, and impair role performance of family members, thereby weakening the overall coordination of the system (15, 16). These disruptions may reduce family adaptability over time. A meta-analysis reported a moderate negative correlation between perceived family adaptability and depressive symptoms, implying that stronger family adaptability was associated with lower levels of depression (17). In dementia care, strengthening family resilience may alleviate caregiver distress by promoting more effective use of internal family resources and strengthening external network support (18).

Family functioning, which reflects the operational quality of the family system, is another key determinant of psychological well-being among family members (19). From the perspective of Structural Family Theory, family organization, role boundaries, and interaction patterns shape how stress is processed within the household. This framework helps explain how family-level mechanisms influence caregiver adaptation and capture the multidimensional nature of family experiences (20). The prolonged burden of caregiving can compromise not only caregivers’ physical and mental health but also their capacity to maintain harmonious relationships with other family members (21).

Among primary caregivers, impaired family functioning is significantly associated with elevated levels of depressive symptoms (22). According to McMaster’s family functioning model, depressive episodes are accompanied by deterioration in multiple functional areas, including problem-solving ability, emotional engagement, and behavioral control (23). As these regulatory domains weaken, families may experience decreased cohesion, impaired role coordination, and reduced organizational stability. This systemic inefficiency limits adaptive coping processes and gradually weakens family resilience under sustained stress (24).

**Figure 1:**
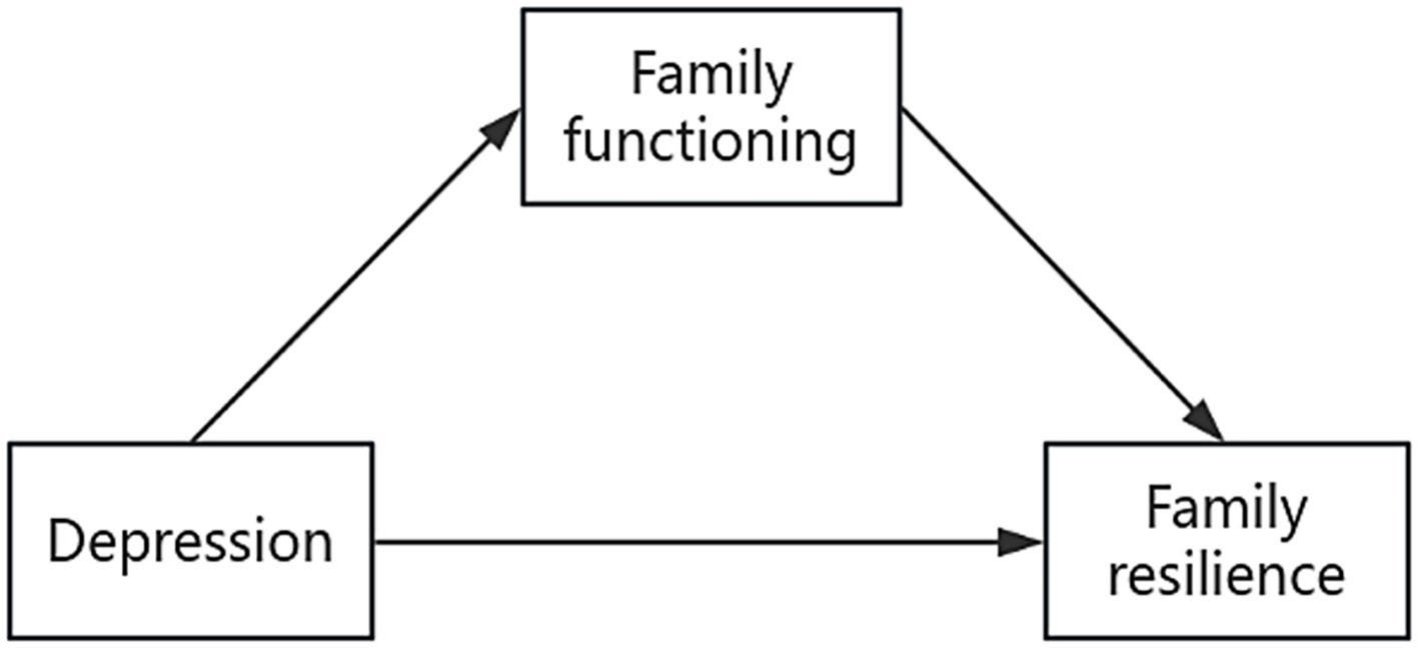
The proposed model elucidates the role of family functioning in explaining the relationship between depression and family resilience.

The interrelationships between depressive symptoms, family functioning, and family resilience can be further explained by family systems theory and family stress models. These frameworks conceptualize family functioning as a dynamic regulatory mechanism through which individual psychological distress influences collective adaptation. Depressive symptoms in caregivers may disrupt interaction patterns and functional balance, thereby impairing the family’s ability to mobilize internal resources and external support. Therefore, family functioning may play a key mediating role, linking caregiver depression to adaptive outcomes at the family level (Figure 1).

The relationship between caregiver depression, family functioning, and family resilience in families of people with dementia remains unclear. Although preliminary evidence suggests that family functioning may mediate the relationship between depressive symptoms and family resilience, systematic empirical investigations in this area are scarce. In the present study, we recruited primary caregivers of individuals with dementia and assessed their levels of depressive symptoms, family functioning, and family resilience.

We further examined whether family functioning mediates the relationship between depression and family resilience, intending to provide both a theoretical foundation and practical insights for improving psychological interventions and strengthening families’ capacity to cope with dementia-related challenges.

## 2 Methods

### 2.1 Participants

This cross-sectional study was conducted in Nanjing, China, between January and December 2024. A total of 514 primary family caregivers of individuals with dementia were recruited through convenience sampling. Participants primarily came from long-established residential communities in Nanjing, which share similar structural and demographic characteristics with mature urban areas in many large and medium-sized Chinese cities. The sample broadly reflects the typical socioeconomic characteristics of the older population in Chinese cities.

Inclusion criteria for individuals with dementia were as follows: (1) aged ≥60 years; (2) diagnosed with dementia by a hospital; (3) residing at home for at least 6 months following diagnosis. Exclusion criterion for individuals with dementia: (1) diagnosis of dementia within the previous 6 months.

Inclusion criteria for family caregivers were as follows: (1) aged ≥18 years; (2) a family member serving as the primary caregiver for the individual with dementia; (3) provided informed consent to participate. Exclusion criteria for family caregivers were as follows: (1) employed by the person with dementia; (2) severe physical or mental illness or recent major life events.

### 2.2 Measures

#### 2.2.1 Family resilience

Family resilience was measured using the Chinese version of the Family Resilience Assessment Scale (FRAS-C), which was translated and culturally adapted by Li et al. (25). The scale comprises 32 items across three dimensions and uses a 4-point Likert scale (1–4), with higher scores reflecting greater family resilience. The total possible score ranges from 32 to 128. In the present study, the FRAS-C demonstrated excellent internal consistency (Cronbach’s *α* = 0.954).

#### 2.2.2 Depression

Depressive symptoms among primary caregivers were assessed using the Self-Rating Depression Scale (SDS) (26). The scale was developed by William W. K. Zung to evaluate the severity of depressive symptoms. The SDS consists of 20 items, including 10 positively worded and 10 reverse-scored items. Participants rated each item on a 4-point scale (1–4) based on their subjective feelings during the preceding week. Total scores were interpreted as follows: <53 indicates no depression; 53–62, mild depression; 63–72, moderate depression; and >72, severe depression. In this study, the SDS exhibited good internal consistency (Cronbach’s *α* = 0.905).

#### 2.2.3 Family functioning

Family functioning was evaluated using the General Functioning subscale of the Family Assessment Device (FAD-GF), originally developed by Epstein et al. (23). The full instrument comprises 60 items across six dimensions and one general functioning subscale. Only the 12-item general functioning subscale was used in this study. Items were rated on a 4-point Likert scale (1–4), with higher scores indicating poorer family functioning. In the current sample, the FAD-GF demonstrated good internal consistency (Cronbach’s *α* = 0.903).

#### 2.2.4 Control variables

This study incorporated control variables based on existing literature and theoretical frameworks. At the patient level, we controlled for dementia type, as different types of dementia are associated with differences in disease progression and behavioral symptoms, which can affect caregiver mental health and family stability (27). Chronic comorbidities were included in the model as an indicator of patient frailty, as patient frailty increases the complexity and burden of care (28). Monthly household income was also controlled for to reflect the role of economic resources in accessing external support and alleviating economic stress (29).

At the caregiver level, age and education level were included in the model as indicators of psychological maturity and knowledge resources related to coping abilities (30). Given the known association between care burden and quality (31), we also controlled for self-rated health status. Finally, we included the number of additional caregivers to reflect informal social support, which can alleviate caregiver stress and influence family resilience (32).

### 2.3 Data collection

This study employed a convenience sampling method and was conducted in six districts of Nanjing (Gulou, Xuanwu, Jiangning, Jianye, Qinhuai, and Yuhuatai). Data collection was supported by institutions providing services for families of people with dementia and local caregiver support groups. All procedures followed ethical standards and adhered to the principle of informed consent. Prior to survey administration, trained researchers explained the study’s purpose, procedures, and confidentiality safeguards to participants. A total of 514 questionnaires were distributed, of which 500 were returned with valid responses, yielding a response rate of 97.3%.

The sample size (*N* = 500) is considered sufficient for bootstrapping-based mediation analysis. Previous methodological and simulation studies have shown that, under small to moderate effect conditions, sample sizes between 200 and 500 are generally sufficient to obtain stable estimates of indirect effects (8, 33).

### 2.4 Statistical analysis

All statistical analyses were performed using SPSS version 26.0 and Stata version 18.0. Descriptive statistics were used to summarize demographic characteristics. Categorical variables were presented as frequencies and percentages. Group comparisons were performed using the chi-square (*χ^2^*) test. Partial correlation analyses were conducted to explore relationships among study variables. Mediation analysis was carried out using the bootstrap method, with statistical significance set at *p* < 0.05.

## 3 Results

### 3.1 Demographic characteristics of individuals with dementia

A total of 500 individuals with dementia and their respective primary family caregivers were included in the analysis, with each patient-caregiver dyad representing one family unit. Among the individuals with dementia, 210 (42.00%) were male and 290 (58.00%) were female. The mean age of individuals with dementia was 83.12 ± 8.92 years. In terms of age distribution, 46 (9.20%) were aged 60–69 years, 101 (20.20%) were aged 70–79 years, 236 (47.20%) were aged 80–89 years, and 17 (3.40%) were aged 90 years or older.

With regard to dementia type, 277 (55.40%) were diagnosed with Alzheimer’s disease, 146 (29.20%) with vascular dementia, 28 (5.60%) with mixed dementia, and 49 (9.80%) with other types. Regarding dementia severity, 146 (29.20%) had mild dementia, 166 (33.20%) moderate dementia, and 188 (37.60%) severe dementia.

Monthly income levels were as follows: 76 (15.20%) had no income; 127 (25.40%) had an income of 1–2,500 yuan; 212 (42.40%) had 2,501–5,000 yuan; 46 (9.20%) had 5,001–7,500 yuan; and 39 (7.80%) had an income >7,500 yuan. Detailed characteristics are presented in Table 1.

**Table 1:** Demographic characteristics of people with dementia.

Variables	Category	*n* (%)	Family resilience score (mean ± SD)	*t/F*	*P*
Gender	Male	210 (42.00)	100.15 ± 1.14	*t* = 0.410	0.682
	Female	290 (58.00)	99.55 ± 0.94		
Marriage	Married	284 (56.80)	99.63 ± 0.99	*t* = 0.265	0.791
	Not married	216 (43.20)	100.02 ± 1.06		
Chronic disease	None	62 (12.40)	97.85 ± 1.95	*t* = −1.008	0.314
	Yes	438 (87.60)	100.07 ± 0.78		
Age (years)				*F* = 1.827	0.141
	60–69	46 (9.20)	98.37 ± 17.44		
	70–79	101 (20.20)	96.8 ± 17.41		
	80–89	236 (47.20)	100.58 ± 15.49		
	> = 90	117 (23.40)	101.38 ± 15.94		
Educational level				*F* = 1.401	0.222
	Illiterate	177 (35.40)	99.78 ± 15.55		
	Primary school	124 (24.80)	98.1 ± 15.80		
	Junior high School	106 (21.20)	99.15 ± 18.18		
	Senior high school	65 (13.00)	103.15 ± 15.80		
	College	9 (1.80)	108.56 ± 18.30		
	Bachelor or above	19 (3.80)	99.05 ± 12.56		
Type of dementia				*F* = 2.551	0.055
	Alzheimer’s disease	277 (55.40)	100.55 ± 16.39		
	Vascular dementia	146 (29.20)	100.44 ± 15.13		
	Mixed dementia	28 (5.60)	92.18 ± 22.30		
	Other	49 (9.80)	98.02 ± 13.32		
Dementia severity				*F* = 2.015	0.134
	Mild	146 (29.2)	101.97 ± 12.52		
	Moderate	166 (33.2)	99.41 ± 16.69		
	Severe	188 (37.6)	98.45 ± 18.15		
Patient’s income (yuan)/M				*F* = 4.014	0.003
	No income	76 (15.20)	95.55 ± 17.09		
	1–2,500	127 (25.40)	101.93 ± 12.32		
	2,501–5,000	212 (42.4)	98.57 ± 18.08		
	5,001–7,500	46 (9.2)	106.13 ± 17.52		
	>7,500	39 (7.8)	100.36 ± 9.43		
ADL				*F* = 2.028	0.109
	Basic self-care	158 (31.60)	100.84 ± 17.11		
	Moderately disabled	67 (13.40)	99.33 ± 12.07		
	Severely disabled	82 (16.40)	95.93 ± 19.33		
	Completely disabled	193 (38.60)	100.76 ± 15.12		

### 3.2 Demographic characteristics of dementia caregivers of people with dementia

A total of 500 caregivers were included in the study, comprising 230 males (46.0%) and 270 females (54.0%). The mean age of caregivers was 64.35 ± 13.02 years. Regarding age distribution, 13 caregivers (2.6%) were aged ≤39 years, 35 (7.0%) were aged 40–49 years, 124 (24.8%) were aged 50–59 years, 163 (32.6%) were aged 60–69 years, and 165 (33.0%) were aged ≥70 years. In terms of self-rated health status, 51 caregivers (10.2%) reported “very good” health, 165 (33.0%) “good” 166 (33.2%), “fair,” 112 (22.4%) “poor,” and 6 (1.2%) “very poor.”

With respect to their relationship to the individual with dementia, 154 caregivers (30.8%) were spouses, 267 (53.4%) were children, 41 (8.2%) were children-in-law, 13 (2.6%) were siblings, and 25 (5.0%) were other relatives. Regarding caregiving support, 3 caregivers (0.6%) reported no additional help, 182 (36.4%) had one additional caregiver, 178 (35.6%) had two, 78 (15.6%) had three, and 59 (11.8%) had four or more. In terms of monthly household income, 160 caregivers (32.0%) reported earnings below 5,000 yuan, 200 (40.0%) between 5,000 and 10,000 yuan, 103 (20.6%) between 10,000 and 15,000 yuan, 25 (5.0%) between 15,000 and 20,000 yuan, 4 (0.8%) between 20,000 and 25,000 yuan, and 8 (1.6%) reported income exceeding 25,000 yuan. A detailed summary of caregiver demographic characteristics is provided in Table 2.

**Table 2:** Demographic characteristics of dementia caregivers.

Variables	Category	*n* (%)	Family resilience score (mean ± SD)	*t*/*F*	*P*
Gender				*t* = 1.648	0.100
	Male	230 (46.00)	101.09 ± 1.01		
	Female	270 (54.00)	98.70 ± 1.06		
Marriage				*t* = 1.935	0.054
	Married	444 (88.80)	100.30 ± 0.75		
	Not married	56 (11.20)	95.86 ± 2.49		
Age (years)				*F* = 3.064	0.016
	≤39	13 (2.60)	109.31 ± 11.75		
	40–49	35 (7.00)	97.86 ± 17.72		
	50–59	124 (24.80)	100.50 ± 15.05		
	60–69	163 (32.60)	101.68 ± 16.08		
	≥70	165 (33.00)	97.07 ± 16.76		
Education level				*F* = 3.545	0.004
	Illiterate	47 (9.40)	93.60 ± 16.50		
	Primary school	69 (13.80)	95.67 ± 15.12		
	Junior high school	162 (32.40)	99.89 ± 15.53		
	Senior high school	144 (28.80)	102.63 ± 15.74		
	College	40 (8.00)	102.90 ± 19.94		
	Bachelor or above	38 (7.60)	100.61 ± 15.55		
Occupation				*F* = 0.726	0.484
	Employed	98 (19.60)	101.41 ± 15.77		
	Unemployed	62 (12.40)	98.45 ± 14.33		
	Retired	340 (68.00)	99.58 ± 16.68		
Self-rate health				*F* = 2.441	0.046
	Very good	51 (10.20)	103.39 ± 19.16		
	Good	165 (33.00)	101.96 ± 13.96		
	Fair	166 (33.20)	98.33 ± 16.78		
	Poor	112 (22.40)	97.38 ± 16.49		
	Very poor	6 (1.20)	95.83 ± 19.01		
Relationship with the patient				*F* = 1.961	0.099
	Spouse	154 (30.80)	97.22 ± 17.34		
	Child	267 (53.40)	101.31 ± 15.56		
	Child-in-law	41 (8.20)	101.80 ± 13.55		
	Sibling	13 (2.60)	98.85 ± 17.09		
	Other	25	96.72 ± 18.24		
Other assistance (person)		(5.00)		*F* = 6.197	0.000
	0	3 (0.60)	91.00 ± 18.36		
	1	182 (36.40)	95.79 ± 18.70		
	2	178 (35.60)	100.43 ± 13.85		
	3	78 (15.60)	104.64 ± 14.84		
	≥4	59 (11.80)	104.31 ± 13.26		
Household income (yuan)/M				*F* = 3.769	0.002
	<5,000	160 (32.00)	96.96 ± 14.97		
	5,000–10,000	200 (40.00)	100.30 ± 16.08		
	10,000–15,000	103 (20.60)	100.1 ± 17.29		
	15,000–20,000	25 (5.00)	110.96 ± 16.47		
	20,000 ~ 25,000	4 (0.80)	98.00 ± 15.34		
	>250,000	8 (1.60)	106.38 ± 15.38		

### 3.3 Family resilience in families of individuals with dementia

The mean family resilience score among families of individuals with dementia was 99.78 ± 16.09. Higher levels of family resilience were observed in households where the individual with dementia reported a monthly income and the total household income was ≥5,000 yuan. Additionally, family resilience scores were comparatively higher when the caregiver was aged <39 years, had attained a high school education or above, and was a child or child-in-law of the individual with dementia.

Moreover, caregivers who reported better self-rated health and those receiving assistance from a greater number of additional caregivers showed significantly stronger family resilience. All of these group differences were statistically significant (*p* < 0.05).

### 3.4 Partial correlation analysis of depression, family resilience and family functioning

Partial correlation analysis revealed that family resilience was significantly negatively correlated with depressive symptoms (*r* = −0.399, *p* < 0.001) and family functioning scores (*r* = −0.617, *p* < 0.001). In contrast, family functioning scores were significantly positively correlated with depressive symptoms (*r* = 0.330, *p* < 0.001). Detailed results are presented in Table 3.

**Table 3:** Partial correlation analysis.

Variables	Mean ± SD	Depression	Family resilience	Family functioning
Depression	38.86 ± 11.61	1		
Family resilience	99.80 ± 16.22	−0.399***	1	
Family functioning	22.64 ± 7.065	0.330***	−0.617***	1

***p < 0.001.

### 3.5 Mediation analysis of depression, family resilience and family functioning

After adjusting for patient income, caregiver age, education level, self-rated health status, relationship to the individual with dementia, number of additional helpers, and monthly household income, the mediating role of family functioning in the relationship between depressive symptoms and family resilience was examined.

The results indicated that depressive symptoms significantly positively predicted family functioning (*B* = 0.187, 95% *CI* [0.133, 0.242], *p* < 0.001). Family functioning significantly negatively predicted family resilience (*B* = −1.228, 95% *CI* [−1.393, −1.063], *p* < 0.001). Depressive symptoms significantly negatively predicted family resilience, representing a significant total effect (*B* = −0.521, 95% *CI* [−0.642, −0.400], *p* < 0.001). When family functioning was included as a mediator, the negative predictive effect of depressive symptoms on family resilience remained significant, indicating a direct effect (*B* = −0.291, 95% *CI* [−0.397, −0.186], *p* < 0.001).

**Figure 2:**
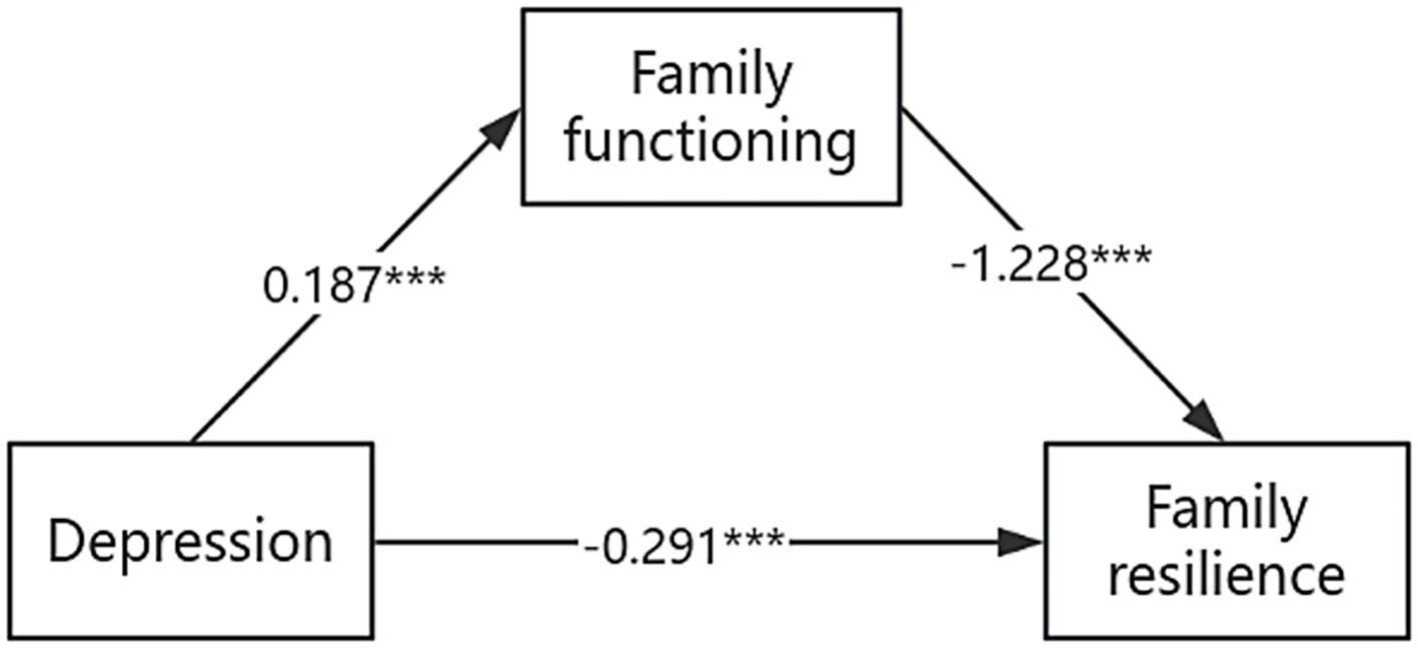
A mediation model illustrating the association between depressive symptoms and family resilience through family functioning. Standardized path coefficients are shown. ***p < 0.001.

Bootstrap analysis with 5,000 resamples confirmed that the indirect effect of depressive symptoms on family resilience through family functioning was significant (*B* = −0.230, 95% bias-corrected bootstrap *CI* [−0.336, −0.142]), as the confidence interval did not include zero. This indirect effect accounted for 44.39% of the total effect, indicating that family functioning played a partial mediating role. The mediation pathway is illustrated in Figure 2, and the results of the bootstrap mediation analysis are summarized in Table 4.

**Table 4:** Bootstrap mediation analysis of the association between depressive symptoms and family resilience via family functioning.

Effect relationship	Estimate	Bootstrap (SE)	Bootstrap (BC 95%CI)	Proportion of total effect (%)
Total effect	−0.518	0.078	[−0.670, −0.363]	
Indirect effect	−0.230	0.049	[−0.325, −0.138]	44.39
Direct effect	−0.288	0.065	[−0.416, −0.160]	55.61

## 4 Discussion

The primary aim of this study was to examine the relationships among depressive symptoms, family functioning, and family resilience in caregivers of individuals with dementia. Our findings provide empirical support for the conceptual model, demonstrating significant interrelationships among these variables. Mediation analysis revealed that caregiver depression is a significant negative predictor of family resilience, indicating that individuals with more severe depressive symptoms have weaker adaptability at the family level. Another important finding is that family functioning serves as a robust mediator in this relationship. The mediating effect accounted for 44.39% of the total effect, suggesting that systemic processes at the family level play a crucial role in explaining how individual psychological distress translates into broader family maladjustment. In short, caregiver depression not only exerts a direct negative impact on family resilience but also indirectly impairs it by undermining the quality of family functioning.

Partial correlation analysis revealed that elevated depressive symptoms were significantly associated with lower family resilience and poorer family functioning, consistent with previous studies (34). Depression is highly prevalent among caregivers of individuals with dementia and manifests not only as persistent low mood but also as social withdrawal, fatigue, irritability, and negative cognitive patterns (16). These symptoms may compromise the quality of family communication, increase interpersonal tensions, and foster maladaptive interaction patterns, ultimately undermining the family’s ability to cope with caregiving-related stress.

It is noteworthy that higher depression scores were associated with higher scores on the family functioning scale, indicating poorer family functioning, a finding consistent with the family stress model. This model posits that when depressive symptoms are compounded by inadequate coping resources and negative cognitive appraisals, the risk of functional disruption within the family system increases (35). Depression may impair caregivers’ ability to fulfill familial roles, contributing to emotional disengagement and heightened intra-family conflict, thereby undermining collaboration and mutual support (36). In turn, dysfunctional family dynamics, characterized by poor communication and low emotional responsiveness, may further intensify psychological stress and reduce access to protective resources such as social support or optimism, thereby exacerbating depressive symptoms in a cyclical fashion.

Conversely, families with better functioning demonstrated significantly stronger resilience, supporting the view that functional family systems play a protective role in the face of adversity (37). High-functioning families are typically characterized by adaptive flexibility, relational closeness, and constructive communication patterns, which collectively enhance the family’s capacity to manage caregiving-related stress and support the psychological well-being of its members (38). Family functioning plays a key mediating role in the positive effects of mutual support behaviors on the family’s capacity for recovery. Such families often demonstrate effective communication, flexible role allocation, and stable emotional support, enabling them to integrate resources and maintain systemic stability under stress.

Importantly, mediation analysis revealed that family functioning partially mediated the relationship between depressive symptoms and family resilience, accounting for 44.39% of the total effect. This suggests that family functioning is not merely a passive outcome influenced by caregiver depression, but serves as an active conduit through which depressive symptoms affect the broader family system. According to Olson’s model of family functioning, resilience is supported by three foundational elements: cohesion, adaptability, and communication (39). Depressive symptoms have the potential to undermine each of these domains by reducing emotional closeness, disrupting flexible role allocation, and impairing communication quality (40). Depressed caregivers may struggle to regulate their emotions, adopt more negative communication patterns, and show reduced motivation for problem-solving, ultimately destabilizing the family system.

This observation aligns with findings from other populations. For instance, Keitner and Miller (41) demonstrated that depression impaired systemic functioning, thereby weakening recovery capacities in military families. Similarly, Shao et al. (37) identified family functioning as a key predictor of family resilience. Collectively, these findings underscore the importance of addressing family-level processes in designing interventions aimed at enhancing caregiver wellbeing and family resilience.

From the perspective of health economics, the mediating role of family functioning can be understood from the perspective of the efficiency of resource allocation within the family. Within the framework of family health production, the family, as a unit, integrates time and material resources to produce health outcomes (42). Caregiver depression has a negative impact on this production system, not only through emotional distress but also by substantially increasing transaction costs within the household (43). When a primary caregiver suffers from depressive symptoms, their cognitive and executive capacities are compromised, leading to friction in decision-making and coordination with other family members (44). This internal friction may elevate the shadow price of care activities and lead to suboptimal allocation of both emotional and material resources (45, 46). Therefore, the weakening of family functioning reflects a decline in the efficiency of resource allocation, which reduces the family ability to mobilize resources to cope with dementia-related crises, thereby weakening the family resilience.

Given these findings, intervention strategies for dementia care in urban communities in China should go beyond individual-level psychological support and incorporate family-system-oriented approaches into the existing primary healthcare and community service infrastructures. At the family level, measures may include psychoeducation, family therapy, or structured communication training to foster emotional closeness, clarify role expectations, and strengthen adaptive problem-solving (7, 47). At the system level, community and healthcare services should be mobilized to provide multi-tiered support, including respite care, psychological counseling, and caregiver skill-building, to relieve burden and promote resilience within dementia care families (48).

Specifically, community health service centers and family doctor contract programs could combine routine screening for depressive symptoms in caregivers with brief assessments of family functioning during regular follow-up visits (49), and refer identified high-risk families to structured family interventions or communication-centered interventions offered through community platforms (50). In addition, long-term care insurance (LTCI) pilot programs and community day care services for the older adults can make caregiver training and respite services reimbursable components, thereby reducing both emotional strain and opportunity costs associated with caregiving (51). Integrating family-centered psychological support into existing urban healthcare and social services systems will further enhance feasibility and sustainability.

## 5 Limitations

This study has some limitations. First, as a cross-sectional study, it cannot draw causal inferences. Furthermore, the relationship between caregiver depression and family dysfunction may be bidirectional. Family dysfunction, such as unequal resource allocation and poor communication, may itself exacerbate depression, suggesting the presence of endogenous factors. Second, the sample is drawn from only one urban area, Nanjing, and may not be representative of caregivers in other regions or socioeconomic backgrounds. Therefore, caution should be exercised when generalizing these findings to rural areas or highly affluent populations. Future research should recruit more diverse and representative samples from different regions and socioeconomic groups, and conduct longitudinal studies to elucidate these relationships and determine their temporal order.

## 6 Conclusion

This study demonstrated that family functioning plays a mediating role in the relationship between depressive symptoms and family resilience among caregivers of individuals with dementia. Depression impacts family resilience both directly and indirectly, with the latter effect transmitted through impairments in family functioning. These findings suggest that enhancing family functioning, particularly in key domains such as communication, cohesion, and adaptability, may help buffer the detrimental impact of depression on caregivers’ resilience. Accordingly, intervention strategies aimed at improving caregivers’ mental health should address not only individual-level depressive symptoms but also system-level improvements in family functioning. Future research could explore longitudinal trajectories and assess the effectiveness of interventions to further validate the mechanisms identified in this study.

## Data Availability

The raw data supporting the conclusions of this article will be made available by the authors, without undue reservation.
